# Contraceptive use, prevalence and incidence of pregnancy and associated factors among women participating in a vaccine preparedness cohort study in Masaka, Uganda, a retrospective secondary analysis

**DOI:** 10.1186/s12978-024-01942-7

**Published:** 2024-12-27

**Authors:** Sylvia Kusemererwa, Sheila Kansiime, Sarah Nakamanya, Elizabeth Mbabazi, Julie Fox, Sheena McCormack, Pontiano Kaleebu, Eugene Ruzagira

**Affiliations:** 1 Medical Research Council/Uganda Virus Research Institute and London School of Hygiene and Tropical Medicine Uganda Research Unit, Plot 51-59, Nakiwogo Road, Entebbe, Uganda; 2 London School of Hygiene and Tropical Medicine, London, UK; 3 King’s College London, London, UK; 4 Medical Research Council Clinical Trials Unit, London, UK

**Keywords:** Contraception, HIV, High risk, Pregnancy, Uganda

## Abstract

**Background:**

HIV prevention trials usually require that women of childbearing potential use an effective method of contraception. This is because the effect of most investigational products on unborn babies is unknown. We assessed contraceptive use, prevalence and incidence of pregnancy and associated factors among women in a HIV vaccine preparedness study in Masaka, Uganda.

**Methods:**

HIV sero-negative women (18–45 years) at high risk of HIV infection identified through HIV counselling and testing (HCT) were recruited between July 2018 and October 2022. Study procedures included collection of baseline socio-demographics and contraceptive use data, quarterly HCT, counselling on and provision of contraceptive methods onsite/through referral, and 6-monthly urine pregnancy tests. Multivariable Logistic and Poisson regression analyses were conducted to determine factors associated with contraceptive use, prevalence, and incidence of pregnancy.

**Results:**

652 (73%) of 891 women reported contraceptive use at baseline. Contraceptive use was higher in women who were in a relationship/married/cohabiting [adjusted odds ratio (aOR) = 1.60; 95% confidence interval (CI) 1.07–2.40] or divorced/separated/widowed [aOR = 1.86; 95% CI 1.24–2.79] versus those that were single, and among women reporting transactional sex [aOR = 2.10; 95% CI 1.16–3.80] versus those who did not. Baseline pregnancy prevalence was 4% (95% CI 3–6%) and lower in women who reported using long-acting contraceptive methods (aOR = 0.17; 95% CI 0.07–0.39) versus women who did not use these methods. A total of 65 pregnancies over 301.3 person-years of observation (PYO), an incidence rate of 21.6/100 (95% CI 16.9–27.5) PYO, higher among younger women (≤ 24 versus 25 + years, adjusted incidence rate ratio = 1.97; 95% CI 1.15–3.40).

**Conclusion:**

We observed a high pregnancy incidence in this cohort. Innovative strategies that promote sustained and consistent use of highly effective contraceptive methods especially for young women will be critical to the success of HIV prevention trials in this and similar populations.

## Plain Language Summary

HIV prevention trials require that women use contraception because the effect of most investigational products on unborn babies in not known. We assessed contraceptive use, incidence of pregnancy and factors associated with contraceptive use among women in a HIV vaccine preparedness study in Masaka, Uganda. HIV sero-negative women (18–45 years) at high risk of HIV infection identified through HIV counselling and testing (HCT) were recruited into the study between July 2018 and October 2022. Study procedures included collection of baseline socio-demographic and contraceptive use data, quarterly HCT, counselling on and provision of contraceptive methods onsite/through referral, and 6-monthly urine pregnancy tests. Regression analyses were conducted to determine factors associated with contraceptive use, prevalence, and incidence of pregnancy. Of 891 enrolled women, 73% reported contraceptive use at baseline. Contraceptive use was almost twice as high in women who were in a relationship/married/cohabiting or divorced/separated/widowed compared to those that were single. Women reporting transactional sex were twice as likely to use contraceptives than women who did not. 4% of the women were pregnant at the time of recruitment. Women using long-acting contraceptives had fewer pregnancies compared to non-users. Incidence of pregnancy was high, especially among younger women (≤ 24 years). Innovative strategies that promote sustained use of highly effective contraceptive methods especially for younger women will be critical to the success of HIV prevention trials.

## Background

Between 2015 and 2019, an estimated 250 million pregnancies (242.7–260.2 million) occurred globally, with 48.3% (121 million) being unintended. Of these unintended pregnancies, approximately 50 million (46.9–53.6 million) were reported in low- and middle-income countries [1]. This poses a challenge and burden to national healthcare systems and minimises the chance of meeting the sustainable development goal (SDG) target of ensuring universal access to sexual and reproductive health care service that include contraception by 2030 [2]. Women at risk of HIV infection (those involved in high-risk sexual behaviour such as transactional sex) in these countries have been found to have high rates of unintended pregnancy, many (11–48%) of which result in induced abortions and related complications [3, 4]. Contraceptive use can significantly reduce unintended pregnancy rates, but in sub-Saharan Africa (SSA), it remains low, with an overall usage rate of only 17% [5]. Among women at high risk of HIV, contraceptive use is also low [6, 7] and varies between 16 and 50% [8]. Reasons for this include influence from male partners, fear of side effects, and health system factors such as access barriers and negative attitudes of healthcare workers towards women at high risk of HIV infection [9].

HIV prevention trials seek to enrol persons at high risk of infection in order to assess the effectiveness of various interventions [10]. These trials require that female participants use contraception because the effects of investigational products on unborn babies are unknown and to reduce time off investigational products due to pregnancy [11, 12]. A high pregnancy incidence results in reduced person-time of follow up, which could affect the statistical power of a trial for per-product efficacy analyses and any analyses to detect product efficacy by gender [13, 14]. Preparedness studies have been used previously to support uptake and effective use of contraception among populations being targeted for HIV prevention trials [15]. These studies have shown that uptake of contraception in these populations can be high [15, 16]. However, only a few preparedness studies have investigated the prevalence and incidence of pregnancy and associated factors among women being prepared to participate in an HIV prevention trial in SSA. Understanding this information would highlight areas on which to focus efforts that support uptake and effective use of contraception during HIV prevention trials thereby minimising time off investigational products.

This study aimed to determine baseline contraceptive use, prevalence and incidence of pregnancy and associated factors (socio-demographic and HIV risk behavioural characteristics) among women in a HIV vaccine preparedness cohort study in Masaka, Uganda. The district is situated in a region with a notably high HIV prevalence (8.1%) [17], making it a crucial area for HIV prevention research.

## Methods

### Study design, setting and population

This was a retrospective secondary analysis of data from the PrEPVacc registration cohort that aimed to identify HIV negative adults (18–45 years) at high risk of HIV infection and prepare them for participation in a phase IIb HIV prophylactic vaccine trial (NCT04066881) at sites in Uganda, Tanzania, Mozambique, and South Africa [18]. In Uganda, the study was conducted between July 2018 and October 2022 at the Medical Research Council/Uganda Virus Research Institute and London School of Hygiene and Tropical Medicine (MRC/UVRI and LSHTM) Uganda Research Unit site in Masaka city, Masaka district, 120 kms southwest of the capital, Kampala.

To identify women at high risk of HIV, we targeted individuals in 10 townships along the Trans-African Highway and 13 fishing villages along Lake Victoria’s shores, across five districts: Masaka, Kalungu, Kyotera, Lwengo, and Lyantonde. Women working in bars, hotels, restaurants, small shops, hair salons, and other local businesses were pre-screened through a door-to-door approach. Trained counselors, supported by community mobilizers, provided HIV counseling and testing, collecting information on age and HIV risk behaviour. Women identified as high-risk were then referred to the research center for further screening and potential enrollment in the study*.*

Women were eligible if they were aged 18 to 45 years, HIV-negative, willing to provide locator information and available for follow-up, and at risk of HIV infection as defined by any of the following: suspected/confirmed sexually transmitted infection or unprotected sex with ≥ 2 partners or unprotected sex with a new partner in the past 3 months or unprotected sex in exchange for money/goods in the past month.

### Contraceptives services

The MRC/UVRI and LSHTM Uganda Research Unit site in Masaka provided contraceptive services to study participants at no cost onsite or through referral to public or private-not-for-profit reproductive care facilities typically located within 1–74 km of the site.

Contraceptive use was defined as use of any of the following short-acting [oral contraceptive pills (combined oral contraceptive and progesterone only pills), male/female condoms] and long-acting [injectable contraceptives (Depo-Provera and medroxy progesterone acetate (MPA)—Sayana Press®), contraceptive implants (Etonogestrel and Levonorgestrel), Copper-T intrauterine contraceptive device (IUCD), and surgical contraceptive methods (bilateral tubal ligation, vasectomy)] methods. Apart from surgical contraceptive methods that were offered through referral, all other methods were offered at the research site. Participants were counselled on contraceptive use by study nurses as per the site standard operating procedures. A study physician assessed each participant to determine the suitability of their preferred contraceptive method, provided advice and recommended alternative methods as necessary and made the prescriptions. At subsequent visits, participants were counselled on continuous and consistent use of their chosen contraceptive method and encouraged to report their experiences. Participants who reported side effects were managed accordingly by study physicians.

### Study procedures

Screening and enrolment procedures were done at a single visit. Screening procedures included obtaining written informed consent, provision of detailed study information, urine pregnancy testing, eligibility assessment, and enrolment of those eligible. Enrolment procedures consisted of collection of locator information, socio-demographics, HIV risk behaviour and current contraceptive use data, and contraceptive counselling.

At quarterly visits, women received contraceptive counselling, and if they chose to, initiated use of their preferred contraceptive method. Every 6 months, women underwent urine pregnancy testing and completed interviewer-administered questionnaires on HIV risk behaviour and contraceptive use.

Recruitment of registration cohort participants into the PrEPVacc trial commenced in December 2020. Participants recruited into the registration cohort after this were typically followed for 3 months or less before joining the trial.

### Laboratory procedures

Beta human chorionic gonadotropin (ßhCG) reagent strips (QuickVue hCG Combo, Quidel Corporation, San Diego, CA 92121, USA and Cypress Diagnostics test hCG Card, Nijverheidsstraat 8 2235 Hulshout, Belgium) were used to perform urine pregnancy tests.

### Statistical analyses

Data collected on case report forms were entered and managed in OpenClinica (Community Edition). Data analyses were conducted in Stata version 16.0 (College Station, TX, USA). The analyses include data collected from 18th July 2018, the first date of enrolment through 3rd October 2022. Categorical data were summarised using percentages while continuous data were summarised using means and standard deviations or medians and interquartile ranges. This analysis had three main outcomes: (a) baseline contraceptive use defined as self-reported use of a form of contraception; (b) pregnancy prevalence defined as number of women who tested positive for urine hCG at baseline divided by the total number of women in the study; (c) pregnancy incidence calculated as total number of pregnancies divided by person-years of observation (PYO).

Key predictors of interest were identified from literature and within the study database, with careful evaluation to avoid strong collinearity. Potential collinearity of predictors was assessed further in descriptive analyses of associations of pairs of variables that were expected to have strong associations by their very nature. Based on this preliminary analysis certain variables would be omitted at multivariable analysis when the other variable was of interest. For prevalence of pregnancy and contraception use at baseline, associations with baseline socio-demographic (age, marital status, level of education, religion and occupation), and behavioural risk characteristics (engaging in transactional sex, type of sexual partner, age of sexual partner, use of alcohol or recreational drugs during sex, number of sexual partners and presence of and STI or its treatment), were assessed using univariable and multivariable logistic regression models. Multivariable model building was conducted using backward stepwise selection. Predictors with a P < 0.2 at univariable analysis were considered at multi-variable analysis. However, only predictors with a P < 0.1 were retained in the multivariable model. The width of the CIs of variables retained in the model were then assessed for imprecision as a result of collinearity. Goodness of fit of the multivariable model was assessed using Hosmer–Lemeshow goodness of fit test.

Univariable and multivariable Poisson regression models were used to assess the associations between baseline socio-demographic, behavioural risk characteristics and incidence of pregnancy. Participants’ follow-up time was limited to either the date of their first positive pregnancy test (if ever pregnant), or the date of their last pregnancy assessment. Participants who were pregnant at baseline were excluded from the pregnancy incidence analyses. A similar modelling approach to that described above (for factors associated with prevalence of pregnancy and contraception use at baseline) was used*.* Goodness of fit of the multivariable model was assessed using the Pearson goodness-of-fit test.

## Results

### Baseline characteristics

A total of 891 women were enrolled. Of these 489 (55%) were ≤ 24 years of age, 467 (52%) were single, 529 (59%) had primary school education or lower, 573 (64%) self-identified as sex workers, and 833 (93%) reported engagement in transactional sex. Most women reported having anonymous/casual sex partners (872, 98%), older (≥ 10 years) sexual partners (682, 77%), and a history of sex after consuming alcohol/using recreational drugs in the past 12 months (525, 59%) (Table 1).

**Table 1:** Factors associated with contraceptive use and pregnancy prevalence among women enrolled in a HIV vaccine preparedness study in Masaka, Uganda (N = 891)

Baseline characteristics	All	Contraceptive use			Prevalence of pregnancy		
	N (%)^a^	n (%)^b^	Univariable analysis	Multivariable Analysis	n (%)^a^	Univariable analysis	Multivariable analysis
			OR (95% CI)^a^	aOR (95% CI)		OR (95% CI)	aOR (95% CI)
Overall	**891 (100)**	**652 (73)**			**37 (4)**		
Age group (years)			**P = 0.001**	P = 0.090		P = 0.815	P = 0.307
≤ 24	489 (55)	336 (69)	0.60 (0.44–0.81)	0.75 (0.54–1.05)	21 (4)	1.08 (0.56–2.10)	0.69 (0.34–1.40)
25 +	402 (45)	316 (79)	Ref	Ref	16 (4)	Ref	Ref
Marital status			**P = 0.001**	**P = 0.003**		P = 0.679	
Single	467 (52)	319 (68)	Ref	Ref	22 (5)	Ref	
In relationship/married/cohabiting	196 (22)	147 (75)	1.39 (0.95–2.03)	1.60 (1.07–2.40)	7 (4)	0.75 (0.31–1.78)	
Divorced/separated/widowed	228 (26)	186 (82)	2.05 (1.39–3.03)	1.86 (1.24–2.79)	8 (4)	0.74 (0.32–1.68)	
Education level			**P = 0.033**			**P = 0.020**	P = 0.061
≤ Primary	529 (59)	401 (76)	Ref		15 (3)	Ref	Ref
≥ Secondary	362 (41)	251 (69)	0.72 (0.54–0.97)		22 (6)	2.22 (1.13–4.33)	1.94 (0.97–3.88)
Religion			P = 0.878			P = 0.743	
Christian	694 (78)	507 (73)	Ref		28 (4)	Ref	
Muslim or other	197 (22)	145 (74)	1.03 (0.72–1.47)		9 (5)	1.14 (053–2.46)	
Occupation			**P < 0.001**	P = 0.053		**P = 0.009**	P = 0.089
Other^c^	137 (15)	89 (65)	Ref	Ref	8 (6)	Ref	Ref
Sex worker	573 (64)	451 (79)	1.99 (1.33–2.99)	1.53 (0.96–2.44)	15 (3)	0.43 (0.18–1.04)	0.58 (0.24–1.45)
Saloon, bar and lodge	181 (20)	112 (62)	0.88 (0.55–1.39)	0.95 (0.58–1.54)	14 (8)	1.35 (0.55–3.32)	1.39 (0.55–3.53)
Reported transactional sex			**P < 0.001**	**P = 0.014**		P = 0.087	
No	58 (7)	27 (47)	Ref	Ref	5 (9)	Ref	
Yes	833 (93)	625 (75)	3.45 (2.01–5.92)	2.10 (1.16–3.80)	32 (4)	0.42 (0.16–1.13)	
Anonymous/casual sexual partners			P = 0.323			P = 0.807	
No	19 (2)	12 (63)	Ref		1 (5)	Ref	
Yes	872 (98)	640 (73)	1.61 (0.63–4.14)		36 (4)	0.78 (0.10–5.97)	
Sexual partner older by ≥ 10 years			P = 0.077			P = 0.360	
No	209 (23)	143 (68)	Ref		11 (5)	Ref	
Yes	682 (77)	509 (75)	1.36 (0.97–1.91)		26 (4)	0.71 (0.35–1.47)	
Used recreational drugs (≤ 3 months)			**P = 0.028**			P = 0.875	
No	738 (83)	529 (72)	Ref		31 (4)	Ref	
Yes	153 (17)	123 (80)	1.62 (1.05–2.49)		6 (4)	0.93 (0.38–2.27)	
Had Sex after consuming alcohol (≤ 12 months)			P = 0.097			P = 0.198	
No	366 (41)	257 (70)	Ref		19 (5)	Ref	
Yes	525 (59)	395 (75)	1.29 (0.96–1.74)		18 (3)	0.65 (0.34–1.25)	
Number of partners (≤ 3 months)			**P < 0.001**	P = 0.094		P = 0.437	
≤ 5	285 (32)	182 (64)	Ref	Ref	14 (5)	Ref	
≥ 6	606 (68)	470 (78)	1.96 (1.44–2.66)	1.39 (0.95–2.03)	23 (4)	0.76 (0.39–1.51)	
Diagnosed/treated for a STI (≤ 3 months)			P = 0.515			P = 0.578	
No	521 (58)	377 (72)	Ref		20 (4)	Ref	
Yes	370 (42)	275 (74)	1.11 (0.82–1.50)		17 (5)	1.21 (0.62–2.34)	
Reported contraceptive use						**P < 0.001**	**P < 0.001**
No	239 (27)	NA	NA	NA	23 (10)	Ref	Ref
Yes (Short-acting)^d^	120 (13)				6 (5)	0.49 (0.20–1.25)	0.51 (0.20–1.30)
Yes (Long-acting)^e^	532 (60)				8 (2)	0.14 (0.06–0.33)	0.17 (0.07–0.39)

Bold values in the first row represent overall totals. Other bold values represent p-values that are less than 0.05

N: Number; OR: Odds ratio; CI: Confidence interval; aOR: adjusted odds ratio; Ref: Reference; NA: not analysed; STI: sexually transmitted infection; NA: Not applicable

^a^Column percentages

^b^Row percentages

^c^Other includes professional/technical worker, sales/service worker, office clerk, student, subsistence fisheries worker etc.

^d^Condom/oral contraceptive

^e^Injectable/Implants/surgical/IUCD

### Contraceptive use and associated factors

Overall, 652 (73%) women reported using at least one contraceptive method at baseline, the commonest methods being: injectable Depo-Provera (300, 34%), implants (181, 20%), IUCDs (29, 3%), oral contraceptive pills 26 (3%); and male condoms (201, 23%). Contraceptive use at baseline was higher in women who were in a relationship/married/cohabiting [adjusted odds ratio (aOR) = 1.60; 95% confidence interval (CI) 1.07–2.40] and those who were divorced/separated/widowed (aOR = 1.86; 95% CI 1.24–2.79) versus those who were single and women who reported transactional sex versus those who did not (aOR = 2.10; 95% CI 1.16–3.80). There was weak evidence that contraceptive use was higher among women whose reported occupation was sex work versus those whose occupation was ‘other’ (aOR = 1.53, 95% CI 0.96–2.44) and among women who reported ≥ 6 sexual partners in the past 3 months versus those who reported less (aOR = 1.39, 95% CI 0.95–2.03). There was also weak evidence that contraceptive use was lower among younger (≤ 24 years) versus older women (25 + years) (aOR = 0.75, 95% CI 0.54–1.05) (Table 1).

The respective proportions of women reporting use of at least one contraceptive method at 6 and 12 months of follow-up were 78% [short acting (53%); long acting (25%)] and 63% [short acting (23%); long acting (40%)]. Approximately 30% of women who were not on long-acting contraceptive methods at baseline took them up by 6 months. Among women who were on a long-acting contraceptive method at baseline, 84% continued use at 6 months of follow up and 63% continued use at 12 months. Uptake and continuation on long-acting methods were largely similar between younger and older women. Switching from a less effective method to a highly effective method was also not different between younger and older women by 12 months (20% versus 23%).

### Pregnancy prevalence, incidence, and associated factors

The overall prevalence of pregnancy at baseline was 4% (95% CI 3–6%). Pregnancy prevalence was lower among contraceptive users, with fewer pregnancies seen among those using long-acting contraceptives versus those who did not (aOR = 0.17 95% CI 0.07–0.39) (Table 1).

A total of 65 pregnancies occurred in 389 women over 301.3 person-years of observation (PYO), an incidence rate of 21.6/100 PYO (95% CI 16.9–27.5). Younger women (≤ 24 years) were more likely to get pregnant than those who were older (adjusted incidence rate ratio = 1.97; 95% CI 1.15–3.40) (Table 2).

**Table 2:** Incidence of pregnancy among women enrolled in a HIV vaccine preparedness study in Masaka, Uganda (N = 389)

Baseline characteristics	N (%)	Pregnancy incidence				
		n (%)	PYO	IR/100 PYO (95% CI)	Univariable analysis	Multivariable analysis
					IRR (95% CI)	aIRR (95% CI)
Overall	**389 (100)**^a^	**65 (17)**^b^	**301.3**	**21.6 (16.9–27.5)**		
Age group					**P = 0.007**	**P = 0.014**
≤ 24 years	214 (55)	46 (22)	162.2	28.4 (21.2–37.9)	2.08 (1.22–3.54)	1.97 (1.15–3.40)
25 + years	175 (45)	19 (11)	139.1	13.7 (8.7–21.4)	Ref	Ref
Marital status					P = 0.264	
Single	206 (53)	35 (17)	169.0	20.7 (14.9–28.8)	Ref	
In relationship/married/cohabiting	97 (25)	22 (23)	78.4	28.0 (18.5–42.6)	1.35 (0.79–2.31)	
Divorced/separated/widowed	86 (22)	8 (9)	53.9	14.8 (7.4–29.7)	0.72 (0.33–1.54)	
Education level					P = 0.111	P = 0.272
≤ Primary	250 (64)	36 (14)	195.5	18.4 (13.3–25.5)	Ref	Ref
≥ Secondary	139 (36)	29 (21)	105.8	27.4 (19.0–39.4)	1.49 (0.91–2.43)	1.32 (0.80–2.17)
Occupation					P = 0.190	
Other^c^	70 (18)	9 (13)	66.4	13.6 (7.1–26.1)	Ref	
Female sex worker	219 (56)	29 (13)	135.6	21.4 (14.9–30.8)	1.58 (0.75–3.33)	
Salon/Lodge/Bar worker	100 (26)	27 (27)	99.4	27.2 (18.6–39.6)	2.00 (0.94–4.26)	
Reported transactional sex					P = 0.559	
No	36 (9)	11 (31)	43.3	25.4 (14.1–45.8)	Ref	
Yes	353 (91)	54 (15)	258.0	20.9 (16.0–27.3)	0.82 (0.43–1.58)	
Has anonymous/casual sex partners					P = 0.192	
No	10 (3)	5 (50)	13.1	38.2 (15.9–91.8)	Ref	
Yes	379 (97)	60 (16)	288.3	20.8 (16.2–26.8)	0.54 (0.22–1.36)	
Sexual partner is older by ≥ 10 years					P = 0.982	
No	90 (23)	17 (19)	78.4	21.7 (13.5–34.9)	Ref	
Yes	299 (77)	48 (16)	222.9	21.5 (16.2–28.6)	0.99 (0.57–1.73)	
Used recreational drugs (≤ 3 months)					P = 0.626	
No	330 (85)	55 (17)	261.2	21.1 (16.2–27.4)	Ref	
Yes	59 (15)	10 (17)	40.2	24.9 (13.4–46.3)	1.18 (0.60–2.32)	
Had Sex after consuming alcohol (≤ 12 months)					P = 0.646	
No	171 (44)	26 (15)	129.0	20.2 (13.7–29.6)	Ref	
Yes	218 (56)	39 (18)	172.3	22.6 (16.5–31.0)	1.12 (0.68–1.84)	
Number of partners (≤ 3 months)					P = 0.412	
≤ 5	148 (38)	36 (24)	151.5	23.8 (17.1–32.9)	Ref	
≥ 6	241 (62)	29 (12)	149.8	19.4 (13.4–27.9)	0.81 (0.50–1.33)	
Diagnosed/treated for an STI (≤ 3 months)					P = 0.250	
No	202 (52)	30 (15)	160.6	18.7 (13.1–26.7)	Ref	
Yes	187 (48)	35 (19)	140.8	24.9 (17.9–34.6)	1.33 (0.82–2.17)	
Reported contraceptive use at baseline					P = 0.370	
No	107 (28)	23 (22)	91.4	25.2 (16.7–37.9)	Ref	
Yes (Short term)^d^	69 (18)	16 (23)	63.0	25.4 (15.6–41.5)	1.01 (0.53–1.91)	
Yes (Long term)^e^	213 (55)	26 (12)	147.0	17.7 (12.0–26.0)	0.70 (0.40–1.23)	

Bold values in the first row represent overall totals. Other bold values represent p-values that are less than 0.05

N: Number of participants with follow up data; PYO: person years of observation; IR: incidence rate; CI: Confidence interval; IRR: incidence rate ratio; aIRR: adjusted incidence rate ratio; Ref: Reference; STI: sexually transmitted infection

^a^Column percentages

^b^Row percentages

^c^Other includes professional/technical worker, sales/service worker, office clerk, student, subsistence fisheries worker etc.

^d^Condom/oral contraceptive

^e^Injectable/Implants/surgical/IUCD

### Pregnancy outcomes

Of the 102 participants who were pregnant at baseline or who became pregnant during follow up, 32 (31%) exited the study while pregnant and 4 (4%) had an unknown pregnancy outcome. Of the remaining 66 participants with known pregnancy outcomes, 46 (70%) had a live birth, 16 (24%) had an induced abortion and 4 (6%) had a spontaneous abortion.

## Discussion

Our study assessed baseline contraceptive use, along with the prevalence and incidence of pregnancy and associated factors among women participating in an HIV vaccine preparedness cohort in Masaka, Uganda. Our findings indicate that contraceptive use was prevalent among the cohort, probably contributing to a low pregnancy prevalence at baseline. Despite this, we observed a high incidence of pregnancy over the study period, pointing to potential gaps in contraceptive accessibility, or consistency of use among participants. These insights underscore important considerations for reproductive health support in HIV prevention efforts.

The reported baseline contraceptive use among women our study was moderately high and comparable to that reported in similar populations [15, 16]. There was an observed drop in contraceptive use at 12 months probably due to selection bias resulting from women who were already using long-acting contraception being recruited into the PrEPVacc trial earlier than those who were not. We found that those who were on short-acting methods at baseline were able to take up long-acting methods in 6 months. Uptake and sustained use of long-acting contraceptives among sexually active women at high risk of HIV infection has been low in previous studies [8]. Further emphasizing the need for contraceptives in this population in the context of HIV prevention trials. One of the major causes for discontinuations in this population has been due to side effects especially menstrual related ones [6, 19].

Overall pregnancy prevalence was similar to what has been reported in related studies [15, 20, 21]. Prevalence could have been low at baseline/enrolment due to women opting not to participate due to pregnancy. Unsurprisingly, women who reported using long-acting contraceptive methods were less likely to be pregnant at baseline compared to those who were not. The pregnancies were those among women reporting use of injectable methods. The latter require action from the user, and have been associated with failure mainly due to delayed repeat injections [22]. Generally, long-acting methods have been reported to be more efficacious with low pregnancy rates documented [23]. In a similar study, pregnancy prevalence was found to be associated with low condom use (short-acting method) and high-risk behavior [21].

The pregnancy incidence among participants in this study was high (21.6 per 100 PYO) and consistent with that reported in similar studies in SSA in which rates ranged from 17.4 to 25.1 per 100 PYO [15, 20, 24]. The observed high pregnancy incidence may be explained by the high proportion of women who reported use of short-acting contraceptive methods—such as condoms and oral contraceptive pills—that have been associated with increased risk of pregnancy among women in SSA [25]. In our study pregnancy incidence among women on long-acting contraception methods was 17.7 per 100 PYO compared to 25.4 per 100PYO in those reporting use of short-acting contraceptives.

The observed high pregnancy incidence among young women (≤ 24 years) has been reported elsewhere and may be explained by several factors. First, younger women in this setting have been shown to have a stronger fertility desire than older women [26] and are less empowered in finding and using contraception [27, 28]. Culturally women in this setting get pregnant to also cement relationships with sex partners [29]. Although younger women were less likely to report contraceptive use than their older counterparts in this study, this difference did not attain statistical significance. Younger women may also be more likely to become pregnant due to peer and family pressure as has been reported elsewhere [20, 24]. They also have increased risk-taking behavior which is of particular concern and probably shows poor understanding and knowledge of contraception or increased dependence on partners’ trust [8].

The finding that contraceptive use was higher among women who were in a relationship, married or cohabiting than in women who were single has been reported in previous studies [27, 30–32]. Compared to single women, those who are in stable relationships may be more motivated to prevent pregnancy either because they have achieved their desired family size or for child spacing [31, 33]. Those who are married are also able to afford contraceptives compared to their counterparts, probably due to stability of the relationship and partner support [27]. The observation that the prevalence of contraceptive use among divorced, separated, or widowed women is higher than that in single women has also been previously reported and may be explained by several factors [31, 32]. It is possible that some of these women were using long-acting contraceptive methods while in a previous long-term relationship and that they continued to use these methods after their partners died or left. Another reason may be that divorced, separated, or widowed women will not want to have children if they have no partner to provide support [34]. We did not have information on outcomes for 35% of the pregnancies observed in the study in part due to some women exiting the study while still pregnant. However, the outcome for almost a quarter of the pregnancies for which data was available, was an induced abortion. This confirms the need for contraception among women at high risk of HIV infection as this population commonly has unintended pregnancies [3, 4].

Our study has some limitations. We did not assess reasons for non-use or uptake of contraception. Nonetheless, we identified factors to be taken into consideration when designing pregnancy prevention strategies for HIV vaccine and other biomedical HIV prevention trials that involve women in SSA. We have previously reported that COVID-19 control measures instituted in 2020–2021 were significantly associated with reduced attendance of follow-up visits in this cohort and loss to follow up [35] combined with early recruitment into the trial. The resulting reduction in person years of follow up may have affected the precision of some of the estimates from the current analyses. Also, since determination of pregnancy was based on a urine test at the study clinic, it is possible that we missed pregnancies among women who missed study visits during this period. Although we promoted contraception, we did not require that women use it as would be expected in a trial evaluating an investigational product. Hence, our findings may not accurately reflect what is to be expected in such a trial. The study was conducted among women at high risk of acquiring HIV. Therefore, these results may not be generalized to women in the general population. Finally, our baseline data on contraceptive use may be susceptible to social desirability bias as we relied entirely on self-report. This is unlike data collected at follow-up visits where contraceptive methods were provided on site or through referral by study staff.

## Conclusions

We found that younger age (≤ 24 years) was associated with low contraceptive use and high pregnancy incidence. Young women at high risk for HIV infection are suitable populations for trials evaluating the efficacy of HIV vaccines and other HIV biomedical prevention products. However, the high pregnancy incidence in such populations and the resultant discontinuation/pause of investigational product use, could negatively impact the power of these trials. Restricting inclusion criteria to having only women on contraception could also result in selection bias in trials. Therefore, strategies to promote use of highly effective contraceptive methods in these populations are urgently needed. These strategies will need to extend beyond the HIV prevention clinical trial field to the wider healthcare settings in order to achieve meaningful impact. Preclinical investigations to support safe use of products in pregnancy are also needed.

## Data Availability

The datasets generated and analysed during the study are available in the LSHTM repository at 10.17037/DATA.00004212. The dataset is managed and made available by the MRC/UVRI and LSHTM Research Unit, in accordance with the data sharing policy and data sharing agreements located at https://apps.mrcuganda.org/mrcdatavisibility/Home/DataShare. The dataset has been collected with a promise to protect the confidentiality of research participants. Limited access to fully anonymised data is permitted for the purpose of research verification, subject to access conditions being met. Researchers interested in the data must apply for access, providing information on the research in which to use the data, the data variables they wish to access, and other relevant information to support their application. Applications will be assessed by the study team at MRC/UVRI and LSHTM Uganda Research Unit, in conjunction with external experts such as the research ethics representative. If the proposed use is allowed under the research ethical conditions, the applicant will be required to sign a Data Transfer Agreement indicating their commitment to store the data securely and use it for the intended purpose only.

## Abbreviations

aOR: Adjusted odds ratio
CA: California
CI: Confidence interval
HIV: Human immunodeficiency virus
IUCD: Intra- uterine contraceptive device
LMICs: Low-and middle-income countries
LSHTM: London School of Hygiene and Tropical Medicine
MPA: Medroxy progesterone HCT: HIV Counselling and testing acetate
MRC/UVRI: Medical Research Council/Uganda Virus Research Institute
PYO: Person years of observation
SDG: Sustainable Development Goal
SSA: Sub-Saharan Africa
ßhCG: Beta human chorionic gonadotropin
TX: Texas
USA: United States of America
